# Assessment of *Tamarix smyrnensis* for Phytoremediation Capacity of Laterite Mine Spoils

**DOI:** 10.3390/plants14030491

**Published:** 2025-02-06

**Authors:** Petroula Seridou, Vasiliki Karmali, Evdokia Syranidou, Konstantinos Komnitsas, Georgios Kolliopoulos, Nicolas Kalogerakis

**Affiliations:** 1 School of Chemical and Environmental Engineering, Technical University of Crete, 73100 Chania, Greece; pseridou@tuc.gr (P.S.); esyranidou@tuc.gr (E.S.); 2 School of Mineral Resources Engineering, Technical University of Crete, 73100 Chania, Greece; vkarmali@tuc.gr (V.K.); kkomnitsas@tuc.gr (K.K.); 3 Département de Génie des Mines, de la Métallurgie et des Matériaux, Université Laval, Québec, QC G1V 0A6, Canada; georgios.kolliopoulos@gmn.ulaval.ca

**Keywords:** air nanobubbles, biochar, halophytes, heavy metals

## Abstract

The phytoremediation potential of the halophytic plant, *Tamarix smyrnensis* (*T. smyrnensis*), was examined in toxic metal spoils assisted by biochar and irrigation by air nanobubbles. The substrate (spoil) used in the present study was derived from areas close to laterite (Ni-containing ores) mines. The efficiency of biochar addition in two rates (5 t/ha and 20 t/ha) to improve microbial properties and stabilize soil aggregates was also examined. Furthermore, the effect of irrigation with air-nanobubble-supplemented water was evaluated for the remediation of toxic metal spoils. The physiological condition of the plant species was investigated in terms of biomass, height, chlorophyll content, and antioxidant enzymes. The alkali and heavy metal accumulation and their distribution in the plant parts were assessed to explore whether toxic metals could accumulate in the root and further translocate to the aboveground tissues. The growth of *T. smyrnensis* was not adversely affected by its cultivation in lateritic spoil, and the highest rate of biochar exhibited a beneficial effect on plant growth in terms of weight (aerial and subterranean biomass). The highest biochar application rate led to significant increases in total chlorophyll content, showing a 97.6% increase when biochar is used alone and a 136% increase when combined with nanobubble irrigation. Remarkably, only when combining irrigation with air nanobubbles and low biochar supplementation did the translocation of the metals from soil to the aboveground tissues occur as the translocation factor was estimated to be greater than unity (TF > 1). The bioconcentration factors remained below 1.0 (BCF < 1) across all treatments, demonstrating limited mobilization from soil to plant tissues despite the application of soil amendments. Finally, the application of nanobubbles increased slightly but not substantially the total uptake of metals, which showed a significant decrease compared to the control groups when the lower dosage of biochar was utilized.

## 1. Introduction

Heavy metals have been proven to exhibit toxic effects on both environmental and human health. They can enter the environment through natural or anthropogenic activities. Natural sources include natural weathering of metal-containing rocks, volcanic eruptions, and soil erosion, while principal anthropogenic sources include fossil fuel combustion, industrial emissions, and discharge, mining, smelting, and agricultural activities like the application of pesticides and phosphate fertilizers [1,2,3,4]. Moreover, heavy metals pose a huge risk to human health even at trace levels since they can enter the body in various ways such as through skin (dermal contact), inhalation, or through the ingestion of contaminated food and water. Owing to their solubility in water they can be absorbed through the intestine and transferred to several organs resulting in their malfunction [5]. For instance, exposure to arsenic has been linked to elevated incidence of many chronic diseases, such as diabetes, cardiovascular diseases, and several types of cancer [6]. Chronic exposure to heavy metals in the environment is a real threat to living organisms; therefore, heavy metal pollution is considered one of the most serious environmental issues worldwide, and there is a dire need to address their remediation since they are highly accumulative and non-biodegradable [7].

Active remediation technologies aim to remove heavy metals ions from a polluted site by means of chemical precipitation, ion exchange, or membrane filtration; however, due to the production of toxic chemical sludge, these techniques are considered as non-eco-friendly [8,9,10]. On the other hand, bioremediation technologies have gained ground thanks to the fact that they are highly effective, cost efficient, and environment-friendly at low metal concentrations [11]. Today, more and more attention has been given to soil contamination, as it has been considered one of the main environmental challenges globally. Soil remediation technologies can be classified as in situ or ex situ, while heavy metals can be removed via a series of physical, chemical, or biological processes [12]. Bioremediation exploiting plant properties and microorganisms has been widely used to remove metals and metalloids from contaminated soils by inducing precipitation, reduction, adsorption, and oxidation through microorganisms (e.g., bacteria, fungi, and algae) [13]. Phytoremediation is a biological process that has been used for many years, which is a low-cost, highly efficient, and green technology. Phytoremediation includes several modes of operation, namely phytoextraction, phytostabilization, phytovolatilization, and rhizofiltration [14,15].

During phytoremediation, plants evolve adaptive strategies to avoid the excess accumulation of metals, which may be potentially harmful. Plants activate mechanisms like cell wall metal binding, the active transport of metal ions into the vacuoles, the chelation of metal ions with proteins and peptides, and complex formation [16]. These mechanisms allow the uptake and distribution of metals within tissues to react according to the need for mineral nutrients in amounts vital for normal growth and avoid the accumulation of non-essential elements and toxic levels of essential elements [17,18]. The accumulation of metals in plant cells is performed in the following order; initially, the mobilization and the adsorption of the metals from the soil is carried out, then their sequestration in the root, and next, their transport into the xylem and to the aerial parts of the plant takes place. Finally, the metal distribution among the leaf cells follows [19]. The rate of metal accumulation is affected by the complex interaction of chelating compounds and metal transporters which is required in each step [19,20]. Among the plant species suitable for metal phytoremediation, halophytes are commonly selected species as they are considered able to cope with stress owing to heavy metals and immobilize them in the root system [21]. Specifically, halophytes have the unique ability to survive in highly saline environments, employing different mechanisms. One of the key strategies is to store high amounts of salt ions in cell compartments, to produce compatible osmolytes and to develop defense systems against oxidative stress. Given these adaptations, it can be assumed that halophytes possess a broad environmental stress tolerance that extends beyond salinity resistance to include the ability to withstand heavy metal toxicity [22]. *T. smyrnensis*, which is grown on the seashore in Crete, Greece, was chosen since its phytoremediation potential in metal-contaminated soils has been investigated in earlier studies [23,24,25,26,27]. However, to our knowledge, no prior studies have examined the capacity of *T. smyrnensis* in laterite mine spoils, which is a substrate unsuitable for plant growth, owing to low nutrient availability, low organic matter, and low water holding capacity [28].

To promote the phytoremediation capacity, the application of various soil amendments may be considered. Primarily, biochar, a solid product from the pyrolysis of waste biomass under low oxygen conditions can improve crop productivity, enable the long-term carbon sequestration in soil [29,30,31], and exhibits significant sorption ability [32,33]. Biochar can increase the pH of soil, exhibits great potential for the precipitation of contaminants, and also supplies and retains nutrients, thus improving soil fertility [34]. Furthermore, the irrigation with water supplemented with air nanobubbles can be utilized as another soil amendment. Nanobubbles (NBs) are gaseous bodies sized less than 1 μm in diameter and exhibit remarkable properties, thanks to their high specific area owing to their small diameter [35,36,37,38]; these properties include a high stagnation time and negligible buoyancy in aqueous solutions leading to their prolonged stability in water [39]. In addition, nanobubbles are characterized by their negative surface charge (zeta potential), immobilizing cations and enhancing the availability of surface nutrient ions [40]. Nanobubble technology can be applied in many fields including agriculture [41,42], as irrigation containing NBs can enhance crop production [43], promote seed germination [44], and also induce a beneficial effect on plant growth [45]. Nanobubble technology has already been integrated in phytoremediation and can enhance heavy metal remediation [46,47]. Experimental studies have indicated that oxygen and air nanobubbles demonstrated maximum efficacy in optimizing soil aeration parameters and enhancing nutrient bioavailability and absorption kinetics. Additionally, nanobubble implementation in soil systems indicated the capacity of nanosized bodies to modify soil–water dynamics through enhanced retention mechanisms and structural reorganization [48,49]. The nanobubble application consequently promotes microbial activity of the rhizosphere and optimizes nutrient absorption by plant roots, thereby contributing to improved soil health [50].

Combining biochar and nanobubble technology presents innovative solutions for agriculture and environmental sustainability. Nanobubbles’ microscopic size facilitates efficient nutrient delivery to plant roots, potentially boosting photosynthetic activity and crop productivity. When combined with biochar, which acts as a reservoir for nutrients and enhances soil structure, this approach can provide a more efficient and sustainable nutrient supply to plants while ameliorating soil quality. Furthermore, this integrated approach supports agricultural carbon sequestration efforts, potentially facilitating carbon-neutral or carbon-negative farming methods. Based on the improved nutrient delivery to plant species and soil quality, this synergistic approach is able to strengthen metal bioaccumulation efficiency as well, paving the way to implement the combination of these two technologies in metal-contaminated soils such as mining sites [51]. While prior studies have extensively documented the effect of biochar and nanobubbles across diverse applications [51,52,53,54,55], there remains a significant knowledge gap regarding the application of their combination in phytoremediation, utilizing halophytes as test plant species. The main objective of this study was to investigate the phytoremediation capacity of *T. smyrnensis* in lateritic mine spoils. The growth of the plant in lateritic spoil in terms of aerial and subterranean biomass, height, as well as the chlorophyll content and the antioxidant enzyme activity was investigated. Additionally, indicators for the phytoremediation capacity such as bioconcentration factor (BCF), translocation factor (TF), and the total uptake (TU) of various metals were determined. The utilization of biochar produced from the pyrolysis of sewage sludge as the soil amendment and irrigation with AirNBs in enhancing the phytoremediation potential of *T. smyrnensis* in lateritic spoil was evaluated. In this study, a synergistic approach that combines biochar amendment and air nanobubble irrigation was introduced for the first time to enhance the phytoremediation of mine spoils. This novel integration uniquely led to increased metal translocation, marking the successful metal extraction from the roots to the upper plant tissues.

## 2. Results

### 2.1. T. smyrnensis Growth

**Figure 1:**
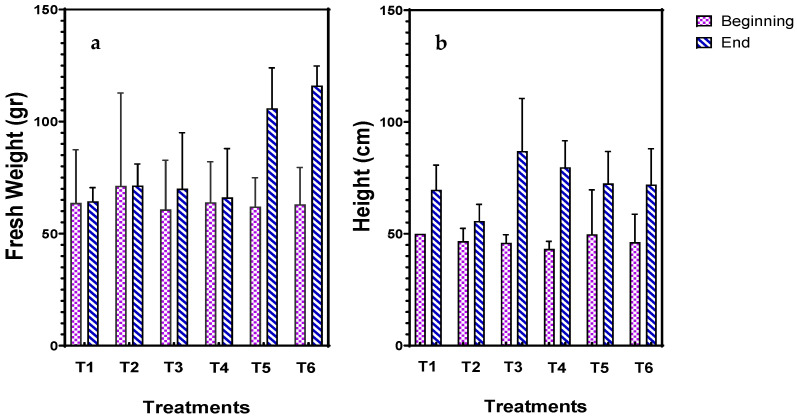
(**a**) Fresh weight (g) and (**b**) height (cm) of *T. smyrnensis* in the beginning and the end of the cultivation period (216 d) in laterite. Treatments have different biochar concentrations (t/ha) and absence (−) or presence (+) of AirNBs: T1 (0/−), T2 (0/+), T3 (5/−), T4 (5/+), T5 (20/−), and T6 (20/+).

In Figure 1 the fresh weight (roots and shoots) and the height of the *T. smyrnensis* before and after cultivation in lateritic spoil are shown. Laterite spoil did not cause substantial toxicity to plants species since no reduction in weight or height was observed. Specifically, in treatments without the addition of biochar (T1 & T2), there was no difference in weight, but a slight increase in the height of plants. The addition of a low rate of biochar (T3 and T4) did not significantly influence the growth of the plant in terms of weight even though a great increase in height was demonstrated. It seems that the addition of a high biochar rate (T5 and T6) substantially increased the weight of *T. smyrnensis* after cultivation in lateritic spoil, an increase that can also be noticed in the height of the plants. The irrigation with NBs did not have any profound impact on the weight or height of the plant species among the six treatments. Overall, *T. smyrnensis* is a tolerant halophyte as its growth in lateritic spoil was not adversely affected.

### 2.2. Chlorophyll Content

**Figure 2:**
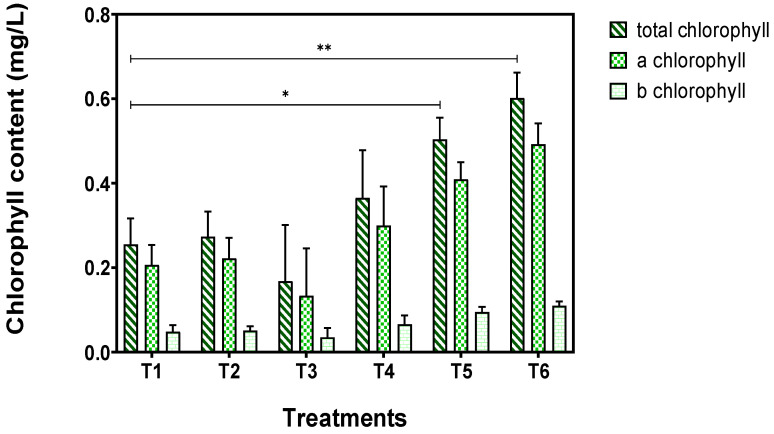
Chlorophyll content of *T. smyrnensis* at the end of the cultivation period (asterisks indicate the level of significance: * for *p* < 0.05 and ** for *p* < 0.01). Treatments have different biochar concentrations (t/ha) and absence (−) or presence (+) of AirNBs: T1 (0/−), T2 (0/+), T3 (5/−), T4 (5/+), T5 (20/−), and T6 (20/+).

Figure 2 exhibits the chlorophyll content of *T. smyrnensis* in all treatments at the end of the cultivation period. The chlorophyll concentration was found to be significantly greater when the highest rate of biochar (20 t/ha) was applied with or without the supplementation of NBs compared to that of the control, implying the positive effects of biochar.

### 2.3. Antioxidant Enzyme Activity

**Figure 3:**
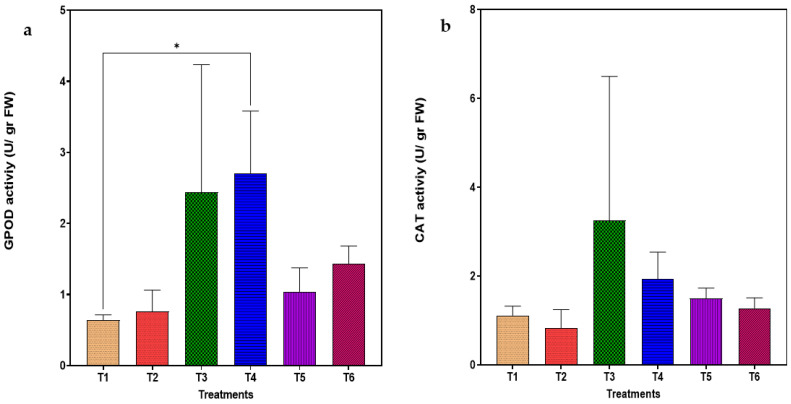
(**a**) Guaiacol peroxidase and (**b**) catalase activity in the roots of *T. smyrnensis* at the end of the cultivation period (asterisk indicates the level of significance: * for *p* < 0.05). Treatments have different biochar concentrations (t/ha) and absence (−) or presence (+) of AirNBs: T1 (0/−), T2 (0/+), T3 (5/−), T4 (5/+), T5 (20/−), and T6 (20/+).

With respect to the guaiacol peroxidase (GPOD) enzyme activity in the roots, it seems that the highest activity was found in treatments T3 and T4 with the lower biochar rate with and without the supplementation of AirNBs. However, there is a statistically significant difference in the treatment with the lower biochar rate and the utilization of AirNBs (T4), as shown in Figure 3. The catalase (CAT) activity was also estimated for all treatments. Even though higher CAT activity was detected in the treatment with a lower biochar rate and irrigated with tap water (T3), there was no statistically significant difference among the treatments.

### 2.4. Bioconcentration Factor (BCF), Translocation Factor (TF), and Total Uptake (TU)

As demonstrated in Table 1, the intervention values for specific metals are listed according to the Dutch soil quality guidelines to probe the pollution levels of the mine spoil. It has been decided to withhold proposing an intervention value for aluminum, manganese, and nutrients [56]. It can be observed that the concentrations of metals Cr, Ni, and Zn in the initial spoil exceeded the intervention values. The metal accumulation was evaluated as well as the concentration of alkali metals in plants since the amount of accumulated nutrients controls the remediation efficiency and the ecological strategy of these plants, and depends on the physiological characteristics of the employed plant species [57]. The bioconcentration factor (BCF) was calculated for various metals in all treatments and is also given in Table 1. The highest BCF was recorded for Na, and it ranged from 14 to 21. The lowest BCF values (≤0.1) were calculated for Al, Fe, and Zn metals. Generally, there were no significant differences due to the addition of biochar or due to irrigation supplemented with AirNBs. In all cases, the BCF values were found to be slightly reduced when the biochar was added. BCF values higher than one indicated a plant species suitable for phytoextraction, as it could extract a higher amount of metals from the soil to plant tissues. The metals in which *T. smyrnensis* exhibited BCF values above unity were Na, K, Ca, and Sr. Regarding Mg, the BCF exceeded one in treatments without the addition of biochar (T1 and T2), while in the remaining treatments, the BCF values were reduced to less than unity.

Apart from the ability of plants to extract the metals in plant tissues, the translocation from the belowground part to the upper part (shoots and leaves) was examined. As shown in Table 2, TF values surpassing one were observed for Na and K in all treatments. Remarkably, there was a great increase over unity in the treatment with the addition of the low biochar rate and the use of irrigation with NBs (T4), except for Mn, which was also found to be increased in this treatment; however, it did not exceed one. It seems that the supplementation of the higher biochar rate did not follow this pattern, as the TF values were similar (i.e., less than one) to those of the control with and without the use of AirNBs. Taken together, these findings suggest the beneficial role of the combination of the two soil amendments for the translocation of metals to the aboveground tissues when a low biochar rate is applied.

Finally, the total uptake of metals in the plants was estimated and is shown in Table 3. The supplementation of AirNBs without the addition of biochar (T2) led to a mild rise, but not a statistically significant one, of metal accumulations in plant tissues compared to those of the control (T1). Additionally, the findings of this study indicated that utilizing the biochar application either individually or alongside nanobubble irrigation yielded a notable reduction in the total uptake of specific metals in plant tissues, which was in some cases statistically significant.

**Figure 4:**
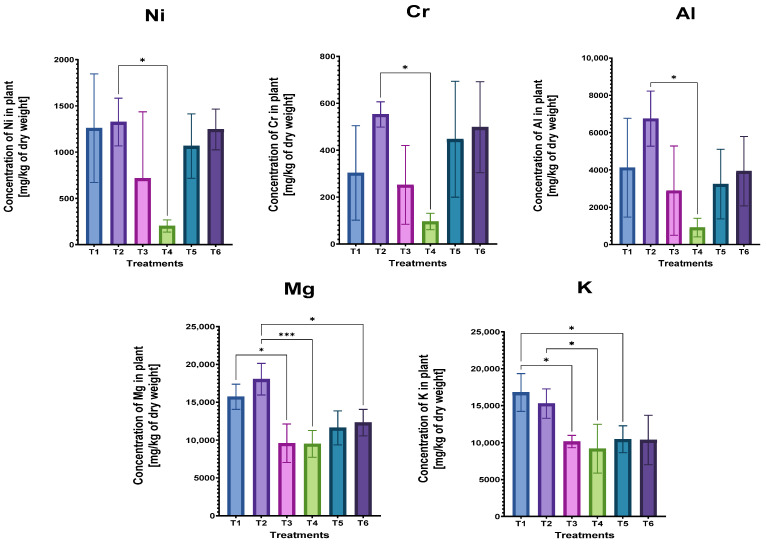
Total uptake (mg/kg plant dry weight) by *T. smyrnensis* for various metals (asterisks indicate the level of significance: * for *p* < 0.05 and *** for *p* < 0. 001). Treatments have different biochar concentrations (t/ha) and absence (−) or presence (+) of AirNBs: T1 (0/−), T2 (0/+), T3 (5/−), T4 (5/+), T5 (20/−), and T6 (20/+).

In fact, a statistical analysis was carried out in order to reveal any significant differences regarding the application of AirNBs and biochar in treatments. Regarding the heavy metals, the uptake in Cr and Ni dropped by almost 80% when a low biochar rate was used, compared to the nanobubble treatment (T2). In addition, Al also exhibited a similar trend as shown in Figure 4. The concentration of the extracted metal was reduced from 6749 to 908 mg/kg of dry weight. The use of biochar significantly decreased the total metal uptake for Mg and K. Specifically, the metal accumulation of Mg was recorded at 15,710 mg/kg and was statistically reduced to 9572 mg/kg (39% reduction) by applying a low biochar rate, while in the case of nanobubble irrigation, the total uptake was reported at 18,035 mg/kg and dropped to 9487 mg/kg and 12,284 mg/kg (a decrease of 47% and 32%), in the low and high biochar rate, respectively. The uptake of K decreased significantly by approximately 40% for both biochar rates, compared to those of the control treatment (T1). The results of this study suggest that nanobubble irrigation combined with the use of biochar resulted in a significant decrease in K uptake for a low biochar rate and Mg uptake for both biochar rates. Hence, it can be concluded that the utilization of the examined soil amendments induced the sharp decrease in the total metal uptake of specific metals.

## 3. Discussion

### 3.1. T. smyrnensis Growth

The growth of *T. smyrnensis* was not negatively affected by its cultivation in lateritic mine spoil. Also, other plant species, specifically Aztec Marigold (*Tagetes erecta* L.) and *Jatropha curcas*, could thrive and grow in mine soil [59,60]. However, others like *Colocasia esculenta* are not tolerant to lateritic soil, losing aboveground biomass [61]. With regard to the weight of *T. smyrnensis*, a great increase of approximately 70% was noticed when a high concentration of biochar (20 t/ha, ~12.6 g/kg spoil) was applied, attributed to the elevated nutrient availability of the biochar that can improve crop growth. The results corroborate previous studies, in which biochar amendment at a similar rate enhanced plant growth [62,63,64]. However, the utilization of nanobubble technology combined with the highest rate of biochar had a negligible effect on plant growth. A previous study demonstrated that the supplementation of oxygen nanobubbles with biochar notably accelerated the root and shoot biomass by approximately 2 and 1.5 times, respectively, for different types of biochar [53]. The growth-promoting effect of biochar application was also confirmed in terms of chlorophyll content, since the addition of biochar at the highest rate substantially augmented the chlorophyll pigments, which was in line with previous studies in the literature [62,65]. The use of biochar combined with AirNBs exhibited further elevation in the chlorophyll pigments compared to those of the control as a statistical difference was observed (*p* < 0.01). Higher concentrations of chlorophyll pigments demonstrated increased light-harvesting capacity for photochemical reactions, thus enhancing photosynthetic efficiency, which serves a critical parameter in optimizing forest ecosystem design and management strategies [66].

### 3.2. Bioconcentration Factor and Total Uptake of Metals

The results regarding the bioconcentration factors showed that there was no remarkable difference among the treatments in terms of metal accumulation. The plants species could accumulate the alkali metals from soil but not the heavy metals, since the bioconcentration factors were estimated to be below unity as seen in Table 1. Polechońska and Klink studied the bioaccumulation of alkali metals by *Phalaris arundinacea* L., and they reported that the bioaccumulation factors exceeded one only for K and Mg and not for Ca and Na [57]. Previous studies have extensively investigated metal bioaccumulation patterns in various halophytic species. Research on *Sesuvium portulacastrum* demonstrated bioconcentration factors (BCFs) exceeding one for both Cd and Cu, although translocation remained notably low [67]. A comparative analysis of *Mesembryanthemum crystallinum* and *Brassica juncea* exposed to 100 ppm NiCl_2_ revealed BCF values of 0.78 and 0.57, respectively [68]. Notably, *Salicornia europaea* exhibited distinctive accumulation characteristics, with BCF values suggesting a potential hyperaccumulator status for nickel (BCF = 0.99), while demonstrating a moderate accumulation capacity for zinc (BCF = 0.10), lead (BCF = 0.12), and cadmium (BCF = 0.14) [69]. Further investigations into metal bioaccumulation in aerial plant tissues of three halophytes, *Suaedamonoica*, *Tamarix indica*, and *Cressa critica*, revealed varying bioaccumulation factors across different metals: Fe (0.018–0.022), Mn (0.030–0.051), Cu (0.075–0.106), Zn (0.107–0.162), Cr (0.085–0.198), and Cd (0.147–0.350) [70]. The studied species exhibited bioaccumulation factors predominantly remaining below unity as demonstrated also in the current study. However, the conventional threshold criterion of BCF > 1 for hyperaccumulator classification may not be definitive, as significant metal accumulation can occur independent of this ratio, highlighting the importance of considering total metal uptake in plant tissues when evaluating phytoremediation potential.

Initially, the treatment of nanobubble irrigation resulted in increased bioconcentration factors across the metal species, while the sodium BCF remained stable and the potassium BCF was reduced. The low-rate biochar application induced a marginal reduction in bioconcentration factors across the majority of metals analyzed. Subsequent irrigation with nanobubble supplementation led to a further decline in bioconcentration values. Higher biochar rates demonstrated reduced bioconcentration values for most metals, while the synergistic application of biochar and nanobubbles showed a negligible effect on bioconcentration factors. The metal accumulation potential of *T. smyrnensis* has been documented [23,71], since the adaptive physiological mechanisms where specialized secretory structures, notably salt glands and trichomes, demonstrate non-selective excretion capacity. This adaptation enables the extrusion of not only sodium and other salt ions but also encompasses the secretion of various toxic elements, thereby contributing to their broad stress tolerance mechanism [72]. However, in this study, the observed bioconcentration factors exhibited markedly low values across all investigated metals, indicating that the phytoextraction efficiency of this species, when cultivated in mine spoil substrates, proved insufficient for effective metal translocation from the contaminated matrix to plant tissues. As anticipated, the observed total uptake values were in accordance with the preceding pattern. The independent application of nanobubble technology yielded a minimal enhancement in total metal extraction efficiency; however, the increment was not statistically significant. Nanobubbles induce the capacity to optimize soil conditions, thereby enhancing microbial activity, organic matter decomposition, and nutrient availability [41]. Previous research has demonstrated that micro/nanobubble-aerated drip irrigation with varying dissolved oxygen concentrations significantly alters the soil microenvironment in the root zone and root system architecture, accompanied by distinct shifts in rhizosphere microbial community structure and endophytic bacterial metabolic profiles [73]. The majority of metals investigated exhibited a reduced uptake under biochar application, which aligns with previous studies reported in the literature [74,75,76]. Similarly, the uptake of the alkali and certain heavy metals further declined when combined with AirNB supplementation. This concurs well with [53], in which it was demonstrated that nanobubbles promoted significant metal stabilization in soil, thereby inhibiting metal accumulation in plants. In the current experimental work, a statistical difference was observed for specific metals, indicating a decreased total uptake in biochar-amended treatments, with and without the application of nanobubble irrigation. This apparent decrease can be justified by the fact that biochar acts as a stabilizing agent for metals in soil; therefore, it can reduce the metal mobility and bioavailability owing to biochar’s metal sorption capacity [77,78]. Indeed, biochar provides strong binding sites for metal ions within its porous structure and alters the pH of soil, thus creating stable metal complexes that minimize metal leaching and consequently decrease metal toxicity in contaminated sites [79,80]. Biochar offers two advantageous effects: carbon sequestration and enhanced crop productivity. The toxic metal concentrations resulting from mining activities create substantial barriers to soil revegetation. Therefore, the selected plants for an effective phytoremediation of mining sites require a multifaceted profile, not only accumulating significant metal concentrations but also demonstrating high biomass yield, environmental adaptability, natural resistance to biological threats, and straightforward cultivation potential. Overall, biochar decreases the bioavailability of metals and metalloids, improves soil structure, fertility dynamics, and revegetation capacity, simultaneously fostering microbial populations, and hence, it is fundamentally essential to investigate the mechanism of biochar-assisted phytoremediation.

### 3.3. Translocation of Metals

The findings of this work indicate that *T. smyrnensis* was able to translocate the alkali and heavy metals in the aboveground part of the plants in the treatments where both soil amendments were supplemented. In particular, the translocation factor was estimated to be over one for all the metals but Mn when irrigation with AirNBs and a low biochar concentration of 5 t/ha (~3.14 g/kg dry spoil) were utilized. In general, the high zeta potential of nanobubbles attracts the positively charged species, thereby facilitating enhanced availability and uptake by plants [47]. Even though several studies have explored nanobubbles as amendments for enhancing metal uptake in plants, they have not specifically examined metal accumulation patterns in aboveground tissues [46,47,81,82]. Previous research has demonstrated that the application of oxygen-nanobubble-loaded biochar inhibited the translocation of copper to plant shoots, which is consistent with the treatment groups where a high biochar concentration was used in this work, since metal accumulation in the upper part was suppressed [53]. It is important to note that the amount of biochar added to the soil in the aforementioned study (10 g/kg dry soil) was comparable to the upper-range concentration reported in our study (12.56 g/kg dry spoil), implying the notable influence of applied biochar dosage on the metal translocation factor. Therefore, it can be concluded that the application of high biochar rates enhanced metal immobilization in the cells of roots through the transformation into low-migration forms, predominantly reducing their accumulation in aerial plant tissues.

Heavy metal contamination induces abiotic stress through enhanced reactive oxygen species (ROS) generation, which triggers cellular oxidative damage via DNA disruption, protein modification, and lipid peroxidation. Plants counter this oxidative stress through the upregulation of antioxidant defense mechanisms, comprising enzymatic and non-enzymatic components. The elevated antioxidant enzyme levels likely facilitated effective reactive oxygen species (ROS) scavenging, consequently maintaining plant health [83]. A significant increase in guaiacol peroxidase activity in the roots was observed in the treatment with the TF above unity, indicating that increased antioxidant activity facilitated the metal transport from roots to the upper part, thereby augmenting the plant’s antioxidative defense mechanisms against mine-spoil-induced oxidative stress. Hence, there is evidence to support the hypothesis that metal-biochar complexes form and can easily migrate from roots to aerial parts, aided by the high electrostatic attraction of AirNBs to cope with the mine-spoil toxicity.

### 3.4. Limitations of This Study

In the current study, a few potential limitations need to be considered. First, this study focused on a specific biochar type and plant species; therefore, further studies which investigate a wider range of diverse plants species as well as biochar types from different feedstocks need to be undertaken to elucidate the phytoremediation performance of various combinations. Additionally, extended longitudinal investigations are imperative to elucidate the remediation efficacy and potential cumulative effects of repeated biochar amendments in the course of time. Furthermore, the complex interactions between biochar amendments and indigenous soil microbiota, which are integral to bioremediation processes, require more detailed mechanistic studies to fully understand their synergistic relationships and functional implications in the remediation process. Another limitation lies in the fact that this study was performed on the laboratory scale; therefore, subsequent field-scale studies are essential to evaluate scalability and the techno-economic feasibility of the implementation of large-scale remediation projects. Field trials are necessary to evaluate the performance of phytoremediation assisted by nanobubbles and biochar under diverse environmental parameters, including climatic variability and heterogeneous soil matrices. With respect to nanobubble systems, their suitability for ecological rehabilitation is evidenced by the widespread commercial availability of economically viable nanobubble generators capable of processing substantial water volumes intended for irrigation purposes. Moreover, biochar is considered a low-cost adsorbent for the removal of various pollutants. The economic feasibility of biochar production is primarily governed by biomass feedstock selection and operational parameters used in its production such as pyrolysis temperature, heating rate, and pressure. Conclusively, a cost-benefit analysis would be beneficial to evaluate the economic viability and societal implications of integrating nanobubble technology and biochar amendments in phytoremediation. Such an analysis would elucidate the financial feasibility and socioeconomic advantages of this combined remediation approach on the commercial scale.

## 4. Materials and Methods

### 4.1. Preparation of Experimental Pots

The *T. smyrnensis* propagation was performed as described by Manousaki et al. [25]. The pot experiment was carried out in the greenhouse of the Technical University of Crete (Chania, Greece), protected from extreme weather conditions, however ensuring full exposure to sun. Initially, the plant cultivation period lasted approximately 2 months to achieve the sufficient biomass of the plants and a mature root system. Then, the plants were divided into 6 experimental groups as shown in Table 4, with homogeneity among the treatments. Each treatment was replicated four times with a total of 24 pots, each containing a plant in 5 L poor Larco ore, which is mine spoil. The characteristics of the lateritic spoil used in this study were detailed in a prior study by our group [58]. The concentration of key metals in the spoil are given here in Table 1 as “initial spoil concentration”. Plants were watered depending on the soil moisture content measured by a moisture meter so that it was maintained at 60% moisture. Plastic trays were placed under each pot to avoid nutrient and metal leachate losses. After a cultivation period of 216 days, entire plants were carefully collected out of the soil and washed with tap water, then rinsed twice with deionized water to remove any dust/dirt. Then, they were separated into roots and shoots for further analysis.

### 4.2. Soil Amendments

Soil amendments were utilized in order to examine whether the phytoremediation capacity could be enhanced. Biochar, produced by sewage sludge obtained from the municipal wastewater treatment plant of the City of Chania (Crete, Greece) was added at two rates (5 t/ha and 20 t/ha) in the specific treatments. Sewage sludge was subjected to slow pyrolysis, which was carried out in a laboratory furnace (LHT08/17 Nabertherm, Lilienthal, Germany) at a temperature of 400 °C. Nitrogen was fed in the furnace at a rate of 100 mL/min, the heating rate was maintained at 10 °C/min, and the retention time of the feedstock was 60 min. The surface area was found to be 55.22 m^2^/g. The detailed properties of the biochar can be found in a previous study conducted by our research group [58]. In addition, the supplementation of air nanobubbles (AirNBs) was performed via the irrigation of the plants. The production of AirNBs was performed by a commercially available MK1 Nanobubbler^TM^ (Fine Bubble Technologies Pty Ltd., Cape Town, South Africa). The device was operated with atmospheric air as the feed gas, producing more than 10^8^ bubbles/mL with a mean diameter of 115 nm.

### 4.3. Bioconcentration Factor (BCF), Translocation Factor (TF), and Total Uptake (TU)

To determine the ability of *T. smyrnensis* to accumulate heavy metals in its biomass, the bioconcentration factor (BCF), translocation factor (TF), and total uptake (TU) were estimated, which are considered very good indicators for heavy metal uptake by plants from soils [59]. BCF is defined as the quantity of heavy metals that is translocated from the soil to the plant and is calculated using Equation (1). It is an index of the ability of the plant to accumulate a particular metal with respect to its concentration in the soil. When the BCF value is higher than 1, the plant is suitable for phytoextraction, and BCF values > 2 are regarded as high values.

(1) $$BCF=\frac{Metal\;Concentration\;in\;Plant\;Tissue\left(Aerial+Subterranean\right)}{Initial\;Concentration\;of\;Metal\;in\;Soil}$$

To evaluate the potential of plants for phytoextraction, the translocation factor (TF) is used. This ratio is an indication of the ability of the plant to translocate metals from the roots to the aerial parts and is calculated using Equation (2). TF values < 1 correspond to metal accumulation in the roots of plants whereas TF values > 1 indicate a transfer to the stems and leaves. A plant with a TF value greater than 1 is considered as a metal accumulator and less than 1 as a metal excluder.

(2) $$TF=\frac{Metal\;Concentration\;in\;Plant\;Tissue\;Aerial\;Parts}{Metal\;Concentration\;in\;Plant\;Tissue\;subterranean\;parts}$$

The total uptake (TU) is the metal concentration in plant tissues in aerial and subterranean parts. It is calculated using Equation (3).

(3) $$\begin{array}{c} TU=Metal\;Concentration\;in\;Plant\;Tissue\;Aerial\;Parts\;+\\ Metal\;Concentration\;in\;Plant\;Tissue\;subterranean\;parts \end{array}$$

### 4.4. Analysis of Plants

#### 4.4.1. Weight and Height Change

At the beginning of the experiment, the fresh weight of the plants was measured. After the experimental period, entire plants were carefully taken out of the soil and washed with tap water, then rinsed twice with deionized water to remove any dust/dirt. Then, they were separated into roots and shoots, and their fresh weights (FWs) were determined. Simultaneously, the height of the shoots was determined before and after the experimental period.

#### 4.4.2. Chlorophyll Content

Leaf samples (0.2 g) were ground in a ceramic mortar with 80% acetone. The absorbance of the supernatant after centrifugation was measured at 663 and 646 nm using a UV–VIS spectrometer to determine the chlorophyll a, chlorophyll b, and total chlorophyll concentrations [84].

#### 4.4.3. Antioxidant Enzymes

For the determination of enzymes, 1 g of fresh samples of leaves and roots was extracted and centrifuged at 16,000× *g* for 25 min. The activity of guaiacol-peroxidase (GPOD) was estimated by monitoring the increase in absorbance due to the oxidation of guaiacol at 470 nm, and the activity of catalase (CAT) was determined, recording the decrease in absorbance as a result of H_2_O_2_ degradation at 240 nm [85,86].

#### 4.4.4. Heavy Metal Analysis

Milled plant samples (1 g) were ashed and dissolved with HNO_3_ (>69%) on a hot plate (~100 °C). The solution was then diluted with ultrapure water and agitated for 24 h. Afterwards, the samples were filtered (0.45 µm; Whatman, Maidstone, UK). In parallel, the total amount of lateritic spoil was collected from the pots, air-dried in plastic bags, and passed once more through a 2 mm mesh-size sieve, and a specific amount of dried laterite spoil was extracted with aqua regia until the sample was completely dissolved and then filtered. The soil and plant metal contents were determined by ICP-MS. Heavy metal concentrations were analyzed using an Agilent 7500 cx ICP-MS at the Technical University of Crete, following US EPA Method 6020. Quality assurance and control protocols included method blanks, control references, and certified reference materials analyzed after every 10 sample injections, along with matrix duplicates in each digestion batch.

### 4.5. Quality Control and Statistical Analysis

Triplicate measurements on the extracts, a measurement of calibration blanks, laboratory reagent blanks, and the standard reference material were employed in order to address data quality control. All data are presented as mean ± standard deviation. Statistical analysis was performed using GraphPad Prism 10 software. Data variation was analyzed with a one-way analysis of variance (ANOVA) at a level of significance of *p* < 0.05.

## 5. Conclusions

This study demonstrated that *T. smyrnensis* was tolerant in lateritic spoil, and even though this substrate is not suitable for plant growth, the addition of soil amendments enhanced the growth of the plant species in terms of weight and height. In addition, the bioconcentration factor values showed that the selected plant species are considered ideal for phytoextraction for the metals Na, K, Ca, and Sr. Mainly, *T. smyrnensis* could not translocate the detected metals to the aboveground tissues except for the treatment where irrigation was supplemented with AirNBs, and a low biochar rate of 5 t/ha was utilized. Interestingly, in this treatment, the translocation factor exceeded unity for all metals, implying the accumulation of the metals from the roots to shoots of the plant. Finally, the total concentration of the metals extracted from the laterite spoil in plant tissues showed a decrease in the case of the biochar addition combined with AirNBs irrigation.

This paper paves the way to clarify the phytoremediation capacity of *T. smyrnensis* cultivated in lateritic mine spoil assisted by soil enrichments that have been widely used for soil amelioration. This study confirms that combining the biochar produced by sewage sludge with irrigation with AirNBs does not have a profound impact on metal accumulation but resulted in, by contrast, a substantial reduction in the total uptake. The addition of biochar at a low rate in conjunction with AirNBs irrigation aided the plant to translocate the metals to the upper plant tissues. The presence of AirNBs alleviated the metal accumulation, and the combination of the soil amendments exhibited a remarkable impact on the metals’ translocation. Overall, even though *T. smyrnensis* was able to grow in the mine spoil, as well as to accumulate the alkali metal, it demonstrated a limited capacity for heavy metal accumulation.

## Figures and Tables

**Table 1:** Bioconcentration factor of *T. smyrnensis* for various metals at different treatments.

Treatment	T1	T2	T3	T4	T5	T6	Initial Spoil Conc. (mg/kg) [58]	Maximum Permissible Level (mg/kg Dry Matter) [56]
**Biochar (t/ha)**	**0**	**0**	**5**	**5**	**20**	**20**		
**AirNBs**	**-**	**+**	**-**	**+**	**-**	**+**		
Heavy metals								
Cr	0.11	0.21	0.10	0.06	0.17	0.19	2648	380
Mn	0.17	0.25	0.22	0.11	0.16	0.19	1793	
Fe	0.04	0.06	0.03	0.01	0.07	0.07	204,503	
Co	0.26	0.41	0.20	0.04	0.32	0.38	185	240
Ni	0.17	0.18	0.10	0.03	0.14	0.17	7520	210
Cu	0.22	0.28	0.14	0.15	0.26	0.20	127	190
Zn	0.07	0.10	0.06	0.09	0.06	0.05	1234	720
Al	0.05	0.08	0.04	0.01	0.04	0.05	79,449	
Ba	0.18	0.30	0.36	0.17	0.20	0.14	83	625
Alkali metals								
Na	21	21	14	14	16	18	403	
Mg	1.3	1.5	0.78	0.77	0.94	1.0	12,286	
K	2.7	2.5	1.7	1.5	1.7	1.7	6102	
Ca	5.9	7.6	6.7	4.0	4.3	4.2	3926	
Sr	1.9	2.4	3.3	1.4	1.4	1.4	42	

**Table 2:** Translocation factor of *T. smyrnensis* for various metals at different treatments.

Treatment	T1	T2	T3	T4	T5	T6
Biochar (t/ha)	0	0	5	5	20	20
AirNBs	-	+	-	+	-	+
Heavy metals						
Cr	0.54	0.33	0.28	2.1	0.20	0.06
Mn	0.49	0.51	0.43	0.89	0.36	0.47
Fe	0.75	0.58	0.83	2.2	0.25	0.56
Co	0.56	0.45	0.61	2.0	0.12	0.05
Ni	0.25	0.48	0.72	1.2	0.15	0.07
Cu	0.51	0.56	0.40	2.1	0.65	0.55
Zn	0.55	0.51	0.26	2.5	0.41	0.59
Al	0.58	0.43	0.64	1.9	0.25	0.09
Ba	0.35	0.51	0.27	1.8	0.33	0.32
Alkali metals						
Na	1.4	1.6	1.0	1.9	2.0	1.8
Mg	0.77	0.87	0.59	1.4	0.94	0.47
K	1.5	1.6	1.4	7.9	1.9	1.3
Ca	0.79	0.66	0.28	1.23	0.67	0.47
Sr	0.89	0.71	0.29	1.1	0.66	0.79

**Table 3:** Total uptake (mg/kg plant dry weight) of *T. smyrnensis* for various metals at different treatments.

Treatment	T1	T2	T3	T4	T5	T6	Initial Spoil Concentration (mg/kg) [58]
Biochar (t/ha)	0	0	5	5	20	20	
AirNBs	-	+	-	+	-	+	
Heavy metals							
Cr	303	553	252	96	447	498	2648
Mn	300	450	393	203	288	345	1793
Fe	8205	11,595	5902	2455	13,589	15,070	204,503
Co	48	75	37	8.0	59	71	185
Ni	1259	1326	717	203	1066	1245	7520
Cu	28	35	18	19	33	25	127
Zn	90	125	79	105	72	66	1234
Al	4121	6749	2886	908	3236	3933	79,449
Ba	15	25	30	14	17	12	83
Alkali metals							
Na	8324	8332	5827	5779	6434	7061	403
Mg	15,710	18,035	9572	9487	11,606	12,284	12,286
K	16,778	15,274	10,137	9173	10,449	10,349	6102
Ca	23,279	29,995	26,285	15,898	16,921	16,352	3926
Sr	80	101	140	57	60	58	42

**Table 4:** Experimental design of *T. smyrnensis* pot experiment in lateritic mine spoil. Treatments have different biochar concentrations (t/ha) and absence (−) or presence (+) of AirNBs.

Treatment	Biochar (t/ha)	Irrigation with AirNBs
T1	0	−
T2	0	+
T3	5	−
T4	5	+
T5	20	−
T6	20	+

## Data Availability

The data presented in this study are available on request from the corresponding author.
